# Polyphasic Characterization of Yeasts and Lactic Acid Bacteria Metabolic Contribution in Semi-Solid Fermentation of Chinese Baijiu (Traditional Fermented Alcoholic Drink): Towards the Design of a Tailored Starter Culture

**DOI:** 10.3390/microorganisms7050147

**Published:** 2019-05-25

**Authors:** Rufei Ma, Lu Sui, Jingsheng Zhang, Jinrong Hu, Ping Liu

**Affiliations:** 1 College of Food Science and Nutritional Engineering, China Agricultural University, Beijing 100083, China; 13007122065@163.com (R.M.); huaxue@cau.edu.cn (J.Z.); hujr@cau.edu.cn (J.H.); 2 Jilin Alcohol Research Institute Co., Ltd, Changchun 130000, China; shuofangssdd@163.com

**Keywords:** yeasts, lactic acid bacteria, semi-solid fermentation, GC–MS, metabolites

## Abstract

Chinese Baijiu is principally produced through a spontaneous fermentation process, which involves complex microorganism communities. Among them, yeasts and lactic acid bacteria (LAB) are important communities. The study examined the isolated strains from fermented grains of Baijiu regarding their activity of α-amylase and glucoamylase, ethanol tolerance, glucose utilization, as well as metabolite production in the process of laboratory-scale sorghum-based fermentation. Selected strains (*Saccharomycopsis fibuligera* 12, *Saccharomyces cerevisiae* 3, and *Pediococcus acidilactici* 4) were blended in different combinations. The influence of selected strains on the metabolic variation in different semi-solid fermentations was investigated by gas chromatography–mass spectrometry (GC–MS) accompanied by multivariate statistical analysis. According to the principal component analysis (PCA), the metabolites produced varied in different mixtures of pure cultures. *S. fibuligera* produced various enzymes, particularly α-amylase and glucoamylase, and exhibited a better performance compared with other species regarding the ability to convert starch to soluble sugars and positively affect the production process of volatile compounds. *S. cerevisiae* had a high fermentation capacity, thereby contributing to substrates utilization. Lactic acid bacteria had a good ability to produce lactic acid. This study attaches importance to the special functions of *S. fibuligera*, *S. cerevisiae*, and *P. acidilactici* in Chinese Baijiu making, and investigates their metabolic characteristics in the process of lab-scale semi-solid fermentation.

## 1. Introduction

Chinese Baijiu, a traditional indigenous fermented alcoholic drink, has gained worldwide acceptance and plays an indispensable role in the Chinese dietary profile [1]. As reported by the China Industry Information Network, in 2017 the annual output of Baijiu was almost 110 million hectoliters with a year-on-year growth of 6.9%; 49.3 million hectoliters were produced in the first half of 2018. Baijiu is traditionally made from cereals such as sorghum, or a mixture of corn, wheat, barley, rice, etc., added with culture starters, and fermented by complex fermentation processes in solid form, semi-solid form, or liquid form [2]. Semi-solid fermentation is conducted under a semi-solid state, which is more efficient in mass transfer and heat transfer, as well as easier to utilize the nutrients in fermented grains when compared to solid-state fermentation [3]. Baijiu contains plentiful flavor compounds like organic acids and esters [4]. These compounds are either directly extracted from the raw materials and ingredients or generated in the alcoholic fermentation process through complex communities of microorganisms, such as yeasts and lactic acid bacteria (LAB) [5]. These microorganisms can significantly affect the quality, safety, and sustainability of Chinese Baijiu. Nevertheless, we barely understand the connection between the microbial structure of fermentation grains and the flavor of Baijiu; poorly-controlled spontaneous fermentation should be replaced by an inoculated fermentation under process control aimed at modernizing the fermentation process of Chinese Baijiu [6].

In the present work, groups of yeasts and bacteria isolated from fermented grains were characterized for their fermentative performances in Baijiu production, and those selected were utilized in lab-scale fermentations.

## 2. Materials and Methods

### 2.1. Screening of Yeast and LAB Isolates for Fermentation

#### 2.1.1. Strain Cultivation

Fifty-one microbial cultures in total, which included 16 isolates of *Saccharomycopsis fibuligera*, 20 isolates of *Saccharomyces cerevisiae*, and 15 isolates of *Pediococcus acidilactici*, were previously isolated by the direct coating method from fermented grains at a facility in Jilin Province. Samples (10 g) were serially diluted 10-fold in sterile peptone physiological salt solution (0.85%) (Oxoid BO0471, Basingstoke, UK), from which aliquots (0.1 mL) were plated on agar medium. Yeasts and LAB were grown on malt extract agar (MEA; Oxoid CM0059) and MRSA (Oxoid CM0361), respectively. All stocks were preserved in 30% glycerol at −80 °C.

#### 2.1.2. Inoculum Preparation

Cultures were cultivated in 10 mL YPD broth (10 g/L yeast extract, 20 g/L peptone, and 20 g/L glucose) for yeast or MRS broth (Oxoid, CM0359) for LAB at 30 °C for 2 days. Sterile peptone physiological salt solution (0.85%) was used to prepare cell suspensions containing 10^8^ cell/mL, based on microscopic count.

#### 2.1.3. Assays of α-Amylase and Glucoamylase Activity

Then 10 mL of growth medium (malt extract broth (MEB) for yeast, and MRS broth for LAB) added with milled sorghum powder (0.1 g), was inoculated with 1 mL of cell suspension (final cell density 10^7^ cell/mL) and grown at 30 °C for 5 days. The cultures were centrifuged at 10,000× *g* for 8 min at 4 °C to obtain the supernatant, which served as crude enzyme [7].

Evaluation of α-amylase and glucoamylase activities was carried out using the EnzyChrom α-Amylase assay kit (Bioassay system, Hayward, CA, USA) and RAMGR3 kit (Megazyme, Wicklow, Ireland), respectively, based on the manufacturers’ instructions.

#### 2.1.4. Glucose Consumption Assay

The preparation of sorghum medium followed a previous report [6]. Briefly, milled sorghum powder (1000 g) was added to deionized water (4 L), which contained moderate thermostable α-amylase solution (1 × 10^4^ U/L). After being boiled for 1 h, the mixture was added with glucoamylase (2.5 × 10^4^ U/L) for saccharification for 4 h at 60 °C. Then the supernatant was obtained and collected as the liquid sorghum extract through centrifugation at 8000× *g* for 6 min. A Leica refractometer (Thermo Fisher Scientific, Pittsburg, PA, USA) was utilized to measure the concentration of sugar in sorghum extract, and the final sugar content in the extract was adjusted to 10° Bx by appropriate dilution with water. Then the extract was sterilized for 15 min at 121 °C before use. A 250 mL Erlenmeyer flask, which contained 100 mL of sorghum extract, was inoculated with an aliquot (1 mL) and cultivated at 30 °C for 7 days. Samples (50 mL) were ultra-sonicated for 30 min at 0 °C, followed by centrifugation for 5 min at 4 °C. The supernatant was collected to measure the concentration of reducing sugars, which was mainly glucose in the sorghum extract. Glucose was determined according to the method of 3,5-dinitrosalicylic acid (DNS) as described in previous study [8].

#### 2.1.5. Ethanol Tolerance Assay

The spot test was performed to investigate the ethanol tolerance of all strains based on Kim’s study [9]. The strains were cultivated in MRS broth for LAB and MEB for yeast to an OD_600_ of 1, followed by ten-fold dilution with a sterile physiological salt solution (0.85%, *w*/*v*). Then each suspension (1 µL) was spotted onto MRSA or MEA plates, which both contained ethanol with different concentrations (*v*/*v*, 0%, 4%, 8%, and 10%). To account for ethanol evaporation, all groups except for the control (without ethanol) had 1% (*v*/*v*) more ethanol added. After inoculation the plates were sealed with parafilm, followed by incubation for 2 days at 30 °C. The calculation of ethanol tolerance depended on the colony size (diameter). Each strain was plated in duplicate.

### 2.2. Fermentation Trials

#### 2.2.1. Inoculum Preparation

Selected strains were cultivated at 30 °C for 2 days in 10 mL of MRS broth for LAB or MEB for yeast. The cell pellets in 1 mL of the culture were obtained through centrifugation at 3000× *g* for 10 min, and then suspended in a sterile physiological salt solution (0.85%, *w*/*v*) to a final density of 10^8^ CFU/mL by microscope count.

#### 2.2.2. Preparation of Semi-Solid Fermentation Medium

One hundred grams of milled sorghum powder added with hot water (60 mL, 80 °C), was then soaked for 60 min. Afterwards the mixture was steamed in an autoclave at 100 °C for 30 min. Then the resulting sorghum paste was obtained and added with sterile cold water (240 g/100 g paste; 20 °C) and cooled down to room temperature before use.

#### 2.2.3. Lab-Scale Fermentation Trials

A sterile 250 mL Erlenmeyer flask containing 100 g of fermentation medium was closed using a water lock. Four independent fermentation trials were performed with different strain mixtures. All these fermentations were inoculated to a final density of 10^6^ cells/mL, and there were three biological replicates for each fermentation. The inocula were *S. fibuligera* 12 (Sf), *S. fibuligera* 12/*S. cerevisiae* 3 (SS), *S. fibuligera* 12/*P. acidilactici* 4 (SP), and *S. fibuligera* 12/*S. cerevisiae* 3/*P. acidilactici* 4 (SSP), always at a ratio of 1:1 for the double culture and 1:1:1 for the triple culture. All the fermentations were incubated for 20 days at 28 °C.

### 2.3. Detection of Acetic Acid and Lactic Acid Content

The contents of acetic acid and lactic acid in the fermentation culture were determined using high performance liquid chromatography (HPLC) with refractive index detector (RID). A Bio-Rad 87H column (Bio-Rad, Hercules, CA, USA) was used and eluted with 5 mM H_2_SO_4_ as a mobile phase at column temperature of 60 °C and a flow rate of 0.6 mL/min.

### 2.4. GC–MS Analysis of Volatile Compounds

The headspace solid-phase microextraction combined with gas chromatography-mass spectrometry (HS-SPME–GC–MS) was used to analyze the flavor compounds in fermentation culture based on previous approaches with minor modification [10]. Then 10 mL of each fermentation culture was added into a 20 mL headspace bottle fitted with a screw cap and a silicon septum, and then mixed with 2 g sodium chloride and 200 μL internal standard (0.0179 g/mL n-butyl acetate). Samples were pre-equilibrated for 20 min at 50 °C. Subsequently, the SPME fiber was inserted into the head space for 60 min at the same temperature. The flavor compounds were extracted with a 50/30 μm divinylbenzene/carboxen/poly(dimethylsiloxane) (DVB/CAR/PDMS) coated fiber (Supelco, Bellefonte, PA, USA).

GC–MS analysis was performed by an Agilent 7890B GC with an Agilent 5977B mass selective detector (Agilent Technologies, Palo Alto, CA, USA), and a DB-Wax capillary column (30 m × 0.25 mm, 0.25 μm; J&W Scientific, Folsom, CA, USA) was used. Helium was used as the carrier gas (linear velocity 1 mL/ min). The GC oven temperature program was as follows: the initial temperature was maintained at 40 °C for 5 min and increased at a rate of 5 °C/min to 60 °C, and subsequently was increased from 60 °C at a rate of 10 °C/min to a final temperature of 230 °C, which was maintained for 8 min. Mass spectra were obtained in electron impact (EI) ionization at 70 eV and the spectrum measurement was performed in the *m*/*z* range of 29–500. The solvent elution delay time was set at 3 min. Each sample was analyzed in triplicate.

### 2.5. Statistical Analysis

Each experiment was performed in triplicate. We calculated the averages as well as standard errors of the mean. SPSS version 20.0 (IBM, SPSS Statistics; Chicago, IL, USA) was used for statistical assessment of all data. The difference significance between the mean values was analyzed by a one-way ANOVA (*p* < 0.05). Principal component analysis (PCA) with MetaboAnalyst software (version 4.0, Xia Lab, McGill University, Montreal, QC, Canada) was performed for analyses of the GC–MS data obtained, which was for visualizing the connection between variables and between variables and samples. The output from the principal component analysis included score plots and loading plots, with the former providing an indication of differentiation among the classes from the perspective of metabolome similarity, and the latter describing the cluster separation regarding the classification gained in the score plots.

## 3. Results

### 3.1. Strain Screening for Activity of α-Amylase, Glucoamylase, and Glucose Utilization

**Figure 1:**
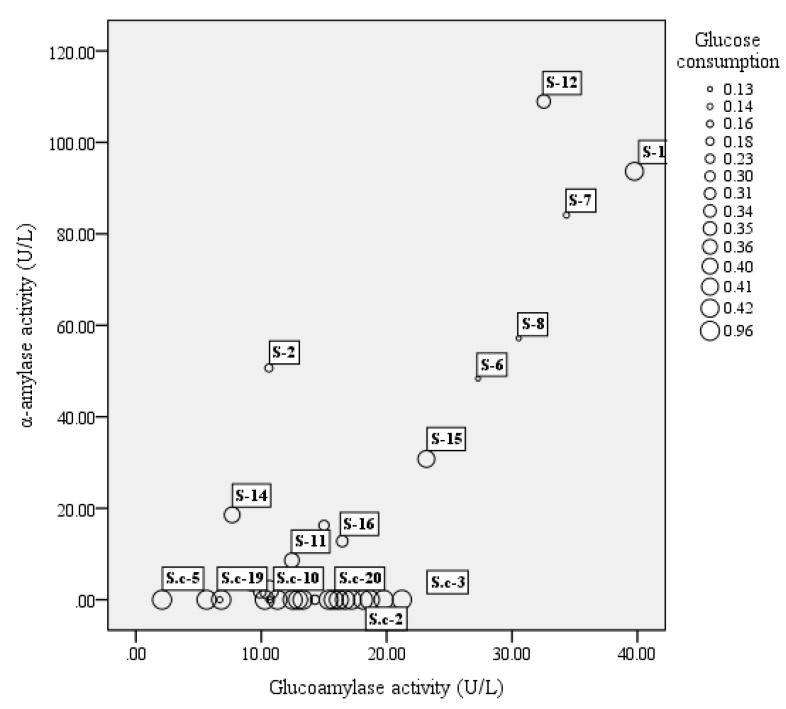
The bubble chart of glucoamylase activity (X-axis), α-amylase activity (Y-axis), and glucose consumption (bubble size) of yeast strains. The results shown are mean of three biological replicates. S = *Saccharomycopsis fibuligera*, S.c = *Saccharomyces cerevisiae*.

**Figure 2:**
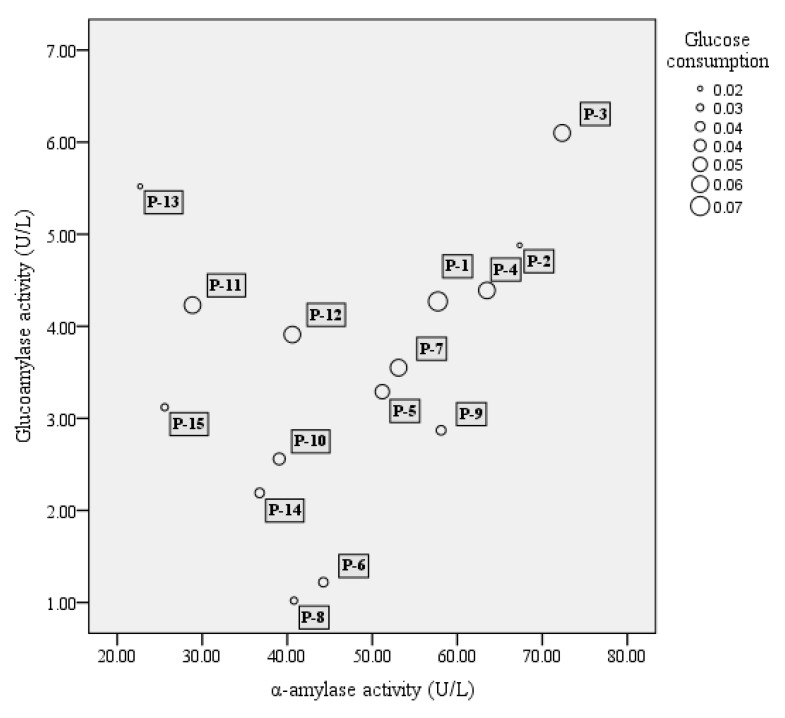
The bubble chart of α-amylase activity (X-axis), glucoamylase activity (Y-axis), and glucose consumption (bubble size) of lactic acid bacteria. The results shown are mean of three biological replicates. P = *Pediococcus acidilactici*.

A total of 51 strains, comprising 16 strains of *S. fibuligera*, 20 strains of *S. cerevisiae*, and 15 strains of *P. acidilactici*, were tested for their enzyme activity including α-amylase, glucoamylase, and glucose consumption. In our study, among 16 tested *S. fibuligera* strains, 13 strains were able to produce both α-amylase and glucoamylase (see Figure 1). Of them S-1 and S-12 showed both high α-amylase and glucoamylase activities. Whereas *S. cerevisiae* showed a lower capacity to generate glucoamylase and α-amylase as compared to *S. fibuligera*, *P. acidilactici* strains generally showed low enzyme activity, with α-amylase activities over the range of 20–70 U/L, and glucoamylase activities less than 7 U/L (see Figure 2).

Of the two yeasts studied, the glucose consumption of *S. cerevisiae* was the highest, reaching 95% (see Figure 1), while the highest glucose consumption of *S. fibuligera* (S-1) was only approximately 42%. In the study, *P. acidilactici* strains generally had low ability in utilization of glucose, with glucose consumption at levels below 10% (see Figure 2).

### 3.2. Strain Screening for Ethanol Tolerance

Table 1 shows the results of ethanol tolerance of microbial isolates (yeasts and LAB). Among the two kinds of yeasts studied, most *S. cerevisiae* strains could tolerate up to 10% (*v*/*v*) ethanol, and only two *S. cerevisiae* strains (S.c-6 and S.c-13) showed growth tolerances on 8% ethanol. None of the tested *S. fibuligera* strains could grow on 10% ethanol, only six of these strains were capable of growing on 8% ethanol. 

Of the 15 strains of *P. acidilactici* studied, only one *P. acidilactici* strain (P-4) was able to grow on 10% alcohol, and P-15 was capable of tolerating 8% ethanol; however, the other *P. acidilactici* strains could only grow on 4% ethanol.

### 3.3. Design of Fermentation Trials with Different Strain Mixtures

Firstly, high abilities of α-amylase, glucoamylase, and glucose utilization were used as criterion for selecting candidate yeast and LAB strains for further fermentation tests. Meanwhile, the selected strains were also compared in terms of their ethanol tolerance. *S. cerevisiae* 3 was chosen according to the above mentioned criterion. *S. fibuligera* 1 and *S. fibuligera* 12 were similar with respect to enzyme ability and glucose consumption, and *S. fibuligera* 12 instead of *S. fibuligera* 1 was chosen considering its slightly higher ethanol tolerance. The strain *P. acidilactici* 4 showed low enzyme activity and glucose consumption, whereas it was also selected due to its higher ethanol tolerance. 

### 3.4. Content of Acetic Acid and Lactic Acid

**Figure 3:**
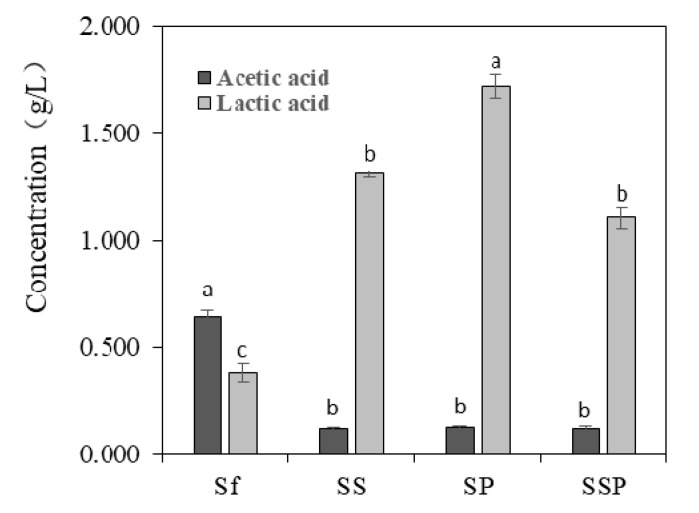
The contents of acetic acid and lactic acid in different fermentation trials. The results shown are means of three replicates. The bars correspond to the estimated average levels. The error bars show standard errors of the mean values. Different letters represent significant difference (*p* < 0.05) of acetic acid or lactic acid content. Sf = *Saccharomycopsis fibuligera* 12, SS = *Saccharomycopsis fibuligera* 12/*Saccharomyces cerevisiae* 3, SP = *Saccharomycopsis fibuligera* 12/*Pediococcus acidilactici* 4, SSP = *Saccharomycopsis fibuligera* 12/*Saccharomyces cerevisiae* 3/*Pediococcus acidilactici* 4.

Acetic acid and lactic acid are two important microbial metabolites produced in the process of Baijiu fermentation. Noteworthy differences could be seen in the amount of acetic acid and lactic acid present in different fermentation trials (Figure 3). *S. fibuligera* proved to have a capacity to produce acid compounds, with the acetic acid and lactic acid concentrations of 0.642 g/L and 0.382 g/L, respectively. The higher contents of lactic acid and lower contents of acetic acid were detected in mixed fermentation with the presence of *S. cerevisiae* or *P. acidilactici*, and particularly in SP fermentations with the content of 1.720 g/L.

### 3.5. The Metabolic Profiles in Different Fermentation Trials

In this study, headspace solid phase microextraction combined with gas chromatography–mass spectrometry (HS-SPME–GC–MS) was used for identification and quantification of the volatile components in different fermentation trials. The grouping of these detected compounds depended on the chemical structures, which consisted of alcohols, esters, acids, aldehydes, ketones, and aromatic compounds (see Table 2).

It was shown that alcohols as the largest class of aroma components were found in all fermentation cultures. They could be produced mainly from sugars in aerobic conditions, as well as by yeasts from amino acids in anaerobic conditions or by chemical reduction of corresponding aldehydes [11]. *S. fibuligera* turned out to be a high generator of higher alcohols. Twelve different higher alcohols were produced, such as isobutyl alcohol, phenethyl alcohol, 1-butanol, and isoamyl alcohol, and the combined concentration reached 71.91 mg/L in Sf fermentations (Table 2). It is known that these by-products are of great importance to the aroma and taste of Baijiu, increasing the sweetness and aftertaste of the liquor [12]. It appeared that higher alcohols were significantly reduced in the mixed fermentations with the presence of *S. cerevisiae* or *P. acidilactici*; although *S. cerevisiae* was expected to produce more alcohol, this difference was mainly accounted for by the contents of phenethyl alcohol, isoamyl alcohol, and 2-butanol. In addition, 1-pentanol was only undetected in Sf fermentation. Furthermore, 2-heptanol and 2-octanol were only detected in SS and SSP fermentation cultures. An interesting result was the higher production of alcohols in triple culture when compared to double culture.

Two acids, including isobutyric acid and caproic acid, were identified in Sf and SP fermentation cultures with low levels. A slight increase was observed in the contents of acids with the presence of *S. cerevisiae*, and butyric acid was found in SS and SSP fermentation cultures. Volatile acids play important roles in the aroma of Baijiu. Components such as caproic acid and octanoic acid increase cheesy, sweaty, and rancid odors; isobutyric acid and butyric acid increase sweaty and acid odors. At the appropriate concentrations, the acids and other aroma compounds jointly form the unique flavor of Baijiu. Moreover, acids as the precursors of esters could significantly affect their production [13].

Esters, as another large group of volatile compounds, were observed in different fermentation cultures. They play important roles in the aroma of not only natural foods (such as fresh fruits) but also fermented foods (like Baijiu). It appeared that ethyl esters turned out to be the richest esters in the fermentation cultures (Table 2). These compounds were mostly metabolites from yeasts, bacteria, or filamentous fungi, as well as formed through esterification of fatty acids with ethanol during the fermentation process [14], proving to be the great contributors to the pleasant floral, fruity, apple-, banana-, and pineapple-like flavors in Baijiu [15]. *S. fibuligera* was also a strong producer of ethyl esters. It produced thirteen different ethyl esters with a combined concentration of 9.61 mg/L in Sf fermentations, such as ethyl hexanoate (fruity), ethyl octanoate (fruity), and ethyl acetate (pineapple), which were the strongest volatile components [16]. The mixed cultures showed significantly lower levels, and ethyl caprate, phenethyl acetate, and ethyl 3-phenylpropionate were only found in Sf fermentations. Some of the esters, such as ethyl lactate, were only found in mixed fermentations, which may be accounted for by high levels of lactate in the presence of *S. cerevisiae* or *P. acidilactici*.

Three aldehydes and six ketones were identified in the cultures. Among them, only 2,5-dimethylbenzaldehyde and 2-octanone were found in all fermentations, indicating that the production of aldehydes and ketones varied among different fermentations. Aldehydes and ketones, generally derived from the degradation of amino acids and lipids in the fermentation process, highly affect the aroma in food and increase the aftertaste of Chinese Baijiu [17].

Six aromatic compounds were identified in the cultures. These are important flavor compounds in various kinds of distilling spirits, which have the characteristics of low content, low threshold, and long retention time. Aromatic compounds mainly come from the synthesis of resolvents of tannin, lignin, protein, etc. in raw materials [18]. It appeared that *S. fibuligera* produced all these components with a combined concentration of 46.09 mg/L in Sf fermentations. Among them, 4-ethyl-2-methoxyphenol (27.75 mg/L), contributing to fruity, sweet, floral, smoky, and rubbery aromas, was highly produced in Sf fermentations. The mixed fermentations considerably reduced the contents of 4-ethyl-2-methoxyphenol as well as 4-ethylphenol, whereas higher production of 4-hydroxy-3-methoxystyrene and 2,3-dihydrobenzofuran were combined with the presence of *S. cerevisiae* considering their significant increase in SS and SSP fermentation cultures.

### 3.6. Statistical Analysis of Flavor Compounds

**Figure 4:**
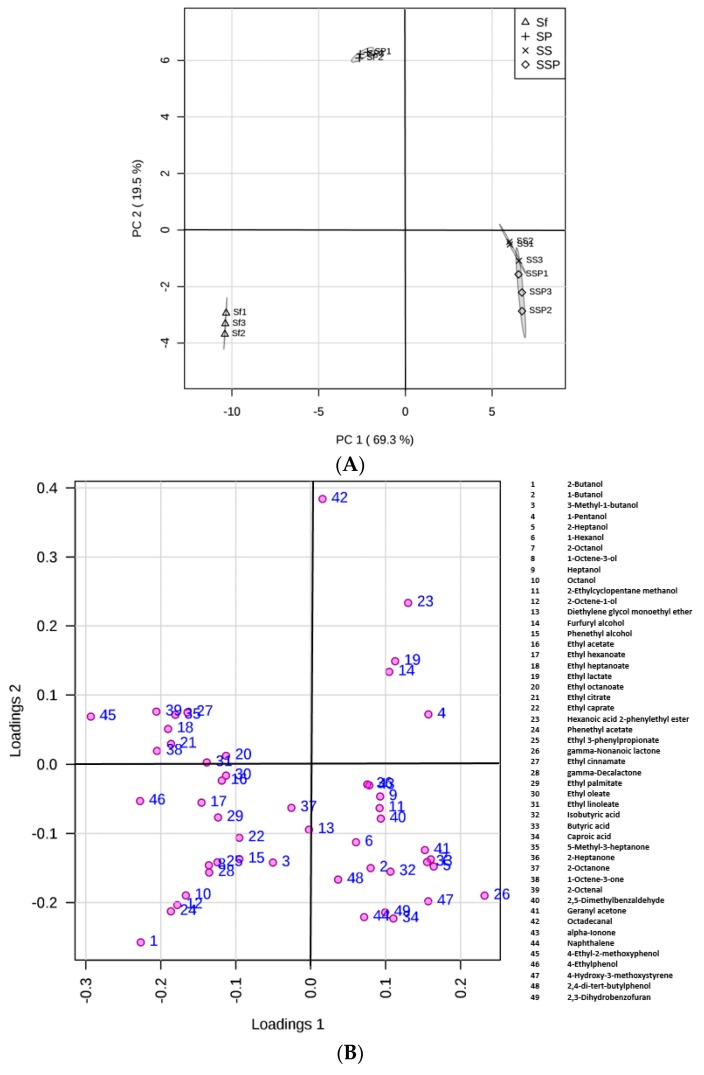
PCA score plot (**A**) and loading plot (**B**) derived from the GC–MS results indicating obvious statistical changes of metabolites in different fermentation trials.

Principal component analysis (PCA) is shown in Figure 4, gaining insight in the nature of multivariate data, evaluating the results of biological interactions, and also checking the experiment reproducibility. High reproducibility of the fermentation procedure was observed with the replicates clustering well. It appeared that these fermentation groups, including Sf, SP, SS, and SSP, could be distinguished (Figure 4A) according to the significant differences of metabolites among them. Additionally, the resulting PCA accounted for 88.8% of the total variance for the first two PCs, with PC1 and PC2 accounting for 69.3% and 19.5, respectively (Figure 4).

Samples taken from the Sf fermentation cultures were on the negative semi-axis of PC1 mainly because of higher levels of alcohols and esters. Alcohols such as 2-butanol, 3-methyl-1-butanol, phenethyl alcohol, and most of the esters were positively correlated with this PC. Samples taken from the SS and SSP fermentation cultures were positioned in the positive phase of PC1, showing the similarity between the two samples but the significant difference from the Sf fermentations in flavor profiles. Compounds such as 2-heptanol, 2-octanol, isobutyric acid, butyric acid, caproic acid, and 4-hydroxy-3-methoxystyrene positioned in the positive phase of PC1, were strongly linked to the SS and SSP fermentations.

Most of these flavor compounds were gathering in the negative phase of PC2, whereas samples taken from the SP fermentations were separated in the positive part, showing that the samples were linked to few aroma components. It appeared that most of the components, especially for higher alcohols and esters, were considerably reduced in SP fermentations when compared to Sf fermentations.

## 4. Discussion

### 4.1. Contributions of Tested Strains in Baijiu Fermentation

#### 4.1.1. *S. fibuligera*

*S. fibuligera* exists in starchy substrates all around the world and is considered as the main amylolytic yeast during the fermentations of indigenous food involving cereals (like rice or sorghum) [19,20,21,22]. It has been reported in several studies that *S. fibuligera* is the only yeast species occurring in different types of Daqu [23]; and the current studies of this species mainly concentrate on brewed wine, such as Chinese rice wine, and the light-flavored Baijiu. These researches revealed that this species usually occupies a dominant position in the starters [24,25]. *S. fibuligera* almost exclusively produces glucoamylase and α-amylase [26], which contribute to glucose accumulation, and it is identical with our results, which showed most tested *S. fibuligera* strains in our study had high enzyme activities, with α-amylase activities over the range of 0–108.96 U/L and glucoamylase activities in the range of 6.69–41.57 U/L (Figure 1). Therefore, *S. fibuligera* usually dominates as the major amylolytic microorganism and plays a vital role at the beginning of the alcoholic fermentation, mainly contributing to degrade the starch or polysaccharides into fermentable, low molecular weight sugars, such as maltotriose, dextrin, and maltose, and these could be subsequently hydrolyzed to glucose, which is the precursor of ethanol and other flavor compounds [27,28]. However, *S. fibuligera* has a lower capacity to utilize glucose when compared to *S. cerevisiae* (Figure 1), and with the accumulation of glucose during fermentation, it is expected that microbial competition could inhibit the survival and growth of *S. fibuligera* [29]. In addition, *S. fibuligera* was both a saccharifying agent and a flavor producer. In the study, 38 flavor components in total were identified and quantified with 12 alcohols, 13 esters, two acids, two aldehydes, three ketones, and six aromatic compounds in single culture of *S. fibuligera* (Sf, Table 2). A study of Wang et al. [30] investigated the fermentation capacity and flavor components of the species isolated from a Maotai-flavor Daqu and the results also indicated that *S. fibuligera* was beneficial for flavor production in fermentation cultures with high contents of phenylethyl alcohol, isoamyl acetate, ethyl acetate, ethyl palmitate, and phenylethyl acetate.

#### 4.1.2. *S. cerevisiae*

*S. cerevisiae* generally predominates during the alcoholic fermentations [29,31,32] as it has a good capacity to grow in strict anaerobic conditions, whereas it is not an abundant yeast species in Daqu. Zheng et al. [33] investigated the microbial diversity in Fen-Daqu, with *S. cerevisiae* undetected through PCR–DGGE analysis and only one isolate of *S. cerevisiae* observed using a culture-dependent approach. *S. cerevisiae* is unable to convert starch to glucose. This was confirmed by our results (Figure 1) showing that most *S. cerevisiae* strains tested showed extremely low enzyme activities. Starch accounts for 65–81% of the full weight of sorghum grains [34]. The growth of *S. cerevisiae* is limited at the beginning of Baijiu fermentation with high contents of starch. However, it has the ability to take over the fermentation process in spite of its low concentration in the initial stage, particularly because of the competitive growth with the presence of fermentable sugars as well as higher alcohol tolerance. This was also proved in our results (Figure 1 and Table 1). This species is expected to grow and flourish, becoming predominant in the liquor fermentation stage as observed during various wine fermentation processes [35,36,37,38]. Additionally, it could be the host yeast species that was recycled in fermentation jars. *S. cerevisiae* is probably considered as the most crucial yeast species during the fermentation process, for its significant contribution to the fermentation rate as well as ethanol production [39]. Thus, the co-culture with the presence of *S. fibuligera* and *S. cerevisiae* could promote ethanol production (data not shown). In addition, we found that the presence of S. cerevisiae was linked to the production of some high alcohols and acids (Figure 4). 

#### 4.1.3. *P. acidilactici*

*P. acidilactici* has been reported to play important roles in many fermented foods, such as wheat sourdough bread [40] and fruit juices [41], mostly contributing to the acidification of raw materials. *P. acidilactici* is also commonly found during Baijiu fermentation, such as Luzhoulaojiao-jiu aged pit mud [42] and fermented grains of sesame-flavor Baijiu [43]. A study of Du et al. [44] investigated the characteristic of carbon utilization of LAB isolated from fermented grains of sesame-flavored Baijiu. The results revealed that most tested LAB had a better ability to use sugars such as dextrin, d-maltose, cellobiose, α-d-glucose, etc., but there were significant differences between different kinds of species. *P. acidilactici* was proved to have a lower capacity to utilize glucose when compared to other substance utilization, such as d-cellobiose, and this might explain the low glucose consumption of most tested *P. acidilactici* (Figure 2). In the study, *P. acidilactici* strain (P-4) were able to tolerate 10% alcohol (Table 1). It is known that many factors can be involved regarding the alcohol tolerance of microbes, such as the changes induced by ethanol about plasma membrane composition, or deactivation of cytosolic enzymes like glycolytic enzymes and ATPase [45]. Other LAB species such as *P. pentosaceus* and *L. plantarum* strains also show obvious alcohol tolerance, for solvent-induced changes about membrane lipid composition [46]. *P. acidilactici* has a good ability to produce the DL-lactic acid in accordance with mechanism of homolactic fermentation, which was observed in our results (Figure 3). This species can also generate bacteriocins, hydrogen peroxide, diacetyl, organic acid, and other antibacterial compounds [47], which contribute to safeguard the microbiological quality of food. However, *P. acidilactici* might contribute little to the production of aroma compounds in the Baijiu fermentation process, which was suggested by the PCA analysis of metabolite profiles (Figure 4).

### 4.2. Interactions of Selected Strains in Baijiu Fermentation

Significant differences in volatile components were found in different fermentations, indicating that co-cultures of different microorganisms influenced the metabolites. Compounds such as 2-butanol, 3-methyl-1-butanol, phenethyl alcohol, and most of the esters were positively correlated with the single culture of *S. fibuligera*. Compounds such as 2-heptanol, 2-octanol, isobutyric acid, butyric acid, caproic acid, and 4-hydroxy-3-methoxystyrene, were strongly correlated with the presence of *S. cerevisiae*. An obvious decrease of the contents of most aroma compounds was observed in the co-culture of *S. fibuligera* and *P. acidilactici*, which might suggest a negative interaction between *S. fibuligera* and *P. acidilactici* for metabolites production. In addition, a higher production of some compounds such as alcohols and aromatic compounds was detected in the triple culture when compared to double culture. This increase might be due to interaction of *S. cerevisiae* and LAB as reported previously [48].

## 5. Conclusions

A significant difference was observed in the outcomes of fermentations (Sf, SP, SS, and SSP), demonstrating that each of the species plays a specific role in different fermentation process. According to statistical analysis, the metabolite profiles were different significantly with the addition or removal of any of the species. The selected strains of *S. cerevisiae* 3, *S. fibuligera* 12, and *P. acidilactici* 4 may have great impact on Chinese Baijiu making. These findings outlined here could contribute to understand more about the interactions among different microbial communities as well as the important function of several primary metabolic products, which would help in improving the effectiveness of added cultures during the process of Baijiu fermentation. However, the characterization of more microorganisms in Baijiu fermentation will require further research.

## Figures and Tables

**Table 1:** Ethanol tolerance of microbial isolates.

Microbial Isolates	Growth on Medium ^1^ Containing Ethanol			
	0	4%	8%	10%
S.c-2, 3, 4, 5, 8, 9, 10, 11, 12, 14, 15, 16, 17, 18, 19, 20	+	+	+	+
S.c-1, 7	+	+	+	W
S.c-6, 13	+	+	+	–
S-3, 4, 6, 10, 12	+	+	+	–
S-14	+	+	W	–
S-1, 2, 5, 7, 8, 9, 11, 13, 15, 16	+	+	–	–
P-4	+	+	+	W
P-15	+	+	W	–
P-1, 2, 3, 5, 6, 7, 8, 9, 10, 11, 12, 13, 14	+	+	–	–

+ = positive, W = weak, – = negative. ^1^ For yeasts, YPDA was used; for lactic acid bacteria, MRSA was used. S.c = *Saccharomyces cerevisiae*, S = *Saccharomycopsis fibuligera*, P = *Pediococcus acidilactici*.

**Table 2:** Concentrations of volatile compounds in different fermentation trials.

Compounds	Concentration (mg/L) ^1^			
	Sf	SP	SS	SSP
**Alcohols**				
2-Butanol	6.60 ± 0.80 ^a^	– ^b^	– ^b^	– ^b^
1-Butanol	0.33 ± 0.06 ^b^	0.17 ± 0.04 ^c^	1.04 ± 0.11 ^a^	0.45 ± 0.05 ^b^
3-Methyl-1-butanol	31.56 ± 1.15 ^a^	13.82 ± 2.94 ^c^	14.41 ± 1.18 ^c^	22.69 ± 3.84 ^b^
1-Pentanol	– ^c^	0.12 ± 0.02 ^b^	0.15 ± 0.05 ^b^	0.36 ± 0.06 ^a^
2-Heptanol	– ^c^	– ^c^	0.13 ± 0.02 ^b^	0.37 ± 0.09 ^a^
1-Hexanol	0.46 ± 0.04 ^bc^	0.36 ± 0.05 ^c^	0.55 ± 0.05 ^b^	0.87 ± 0.17 ^a^
2-Octanol	– ^c^	– ^c^	0.14 ± 0.01 ^b^	0.24 ± 0.04 ^a^
1-Octene-3-ol	1.41 ± 0.33 ^a^	0.20 ± 0.06 ^b^	0.16 ± 0.02 ^b^	0.21 ± 0.07 ^b^
Heptanol	0.20 ± 0.02 ^c^	0.25 ± 0.04 ^bc^	0.38 ± 0.10 ^b^	0.54 ± 0.11 ^a^
Octanol	0.46 ± 0.04 ^a^	– ^b^	– ^b^	– ^b^
2-Ethylcyclopentane methanol	0.19 ± 0.02 ^c^	0.22 ± 0.04 ^bc^	0.31 ± 0.04 ^b^	0.61 ± 0.08 ^a^
2-Octene-1-ol	0.74 ± 0.21 ^a^	– ^b^	– ^b^	– ^b^
Diethylene glycol monoethyl ether	2.10 ± 0.35 ^b^	1.47 ± 0.27 ^bc^	1.14 ± 0.08 ^c^	3.16 ± 0.92 ^a^
Furfuryl alcohol	0.11 ± 0.04 ^c^	0.69 ± 0.17 ^b^	0.28 ± 0.04 ^c^	0.98 ± 0.11 ^a^
Phenethyl alcohol	27.75 ± 0.99 ^a^	8.08 ± 1.80 ^b^	7.98 ± 0.11 ^b^	9.86 ± 0.80 ^b^
**Esters**				
Ethyl acetate	1.32 ± 0.08 ^a^	0.67 ± 0.04 ^b^	0.33 ± 0.05 ^c^	0.42 ± 0.07 ^c^
Ethyl hexanoate	1.07 ± 0.07 ^a^	0.31 ± 0.04 ^b^	0.11 ± 0.04 ^c^	0.19 ± 0.05 ^c^
Ethyl heptanoate	0.42 ± 0.07 ^a^	0.16 ± 0.02 ^b^	– ^c^	– ^c^
Ethyl lactate	– ^d^	0.19 ± 0.02 ^b^	0.07 ± 0.04 ^c^	0.30 ± 0.05 ^a^
Ethyl octanoate	0.39 ± 0.03 ^a^	0.26 ± 0.05 ^b^	0.09 ± 0.02 ^d^	0.16 ± 0.02 ^c^
Ethyl citrate	0.37 ± 0.02 ^a^	0.12 ± 0.02 ^b^	– ^c^	– ^c^
Ethyl caprate	0.06 ± 0.02 ^a^	– ^b^	–^b^	– ^b^
Hexanoic acid 2-phenylethyl ester	– ^c^	0.50 ± 0.05 ^a^	0.19 ± 0.02^b^	0.30 ± 0.05 ^b^
Phenethyl acetate	1.02 ± 0.17 ^a^	– ^b^	– ^b^	– ^b^
Ethyl 3-phenylpropionate	0.12 ± 0.02 ^a^	– ^b^	– ^b^	– ^b^
γ-Nonanoic lactone	– ^c^	– ^c^	1.62 ± 0.00^b^	2.27 ± 0.05 ^a^
Ethyl cinnamate	0.40 ± 0.06 ^a^	0.21 ± 0.01^b^	0.07 ± 0.01^c^	– ^d^
γ-Decalactone	0.18 ± 0.07 ^a^	– ^b^	– ^b^	– ^b^
Ethyl palmitate	2.55 ± 0.30 ^a^	0.84 ± 0.07 ^b^	0.51 ± 0.02 ^c^	0.65 ± 0.11 ^bc^
Ethyl oleate	0.88 ± 0.15 ^a^	0.49 ± 0.12 ^b^	0.12 ± 0.02 ^c^	0.39 ± 0.09 ^b^
Ethyl linoleate	0.83 ± 0.16 ^a^	0.39 ± 0.04 ^b^	0.16 ± 0.02 ^c^	0.16 ± 0.02 ^c^
**Acids**				
Isobutyric acid	0.14 ± 0.04 ^b^	0.09 ± 0.02 ^b^	0.45 ± 0.06 ^a^	0.39 ± 0.09 ^a^
Butyric acid	– ^c^	– ^c^	0.11 ± 0.04 ^b^	0.29 ± 0.08 ^a^
Caproic acid	0.22 ± 0.09 ^b^	0.07 ± 0.03 ^b^	0.65 ± 0.13 ^a^	0.82 ± 0.11 ^a^
**Aldehydes and Ketones**				
2-Octenal	0.70 ± 0.13 ^a^	0.28 ± 0.07 ^b^	– ^c^	– ^c^
2,5-Dimethylbenzaldehyde	0.67 ± 0.07 ^c^	0.69 ± 0.13 ^c^	1.30 ± 0.10 ^b^	1.66 ± 0.32 ^a^
Octadecanal	– ^c^	1.11 ± 0.30 ^a^	0.17 ± 0.04 ^b^	– ^c^
5-Methyl-3-heptanone	0.32 ± 0.09 ^a^	0.16 ± 0.02 ^b^	– ^c^	– ^c^
2-Heptanone	–^b^	–^b^	0.12 ± 0.02 ^a^	–^b^
2-Octanone	0.24 ± 0.04 ^ab^	0.19 ± 0.05 ^b^	0.13 ± 0.02 ^b^	0.28 ± 0.04 ^a^
1-Octene-3-one	0.71 ± 0.15 ^a^	0.16 ± 0.02 ^b^	– ^c^	– ^c^
Geranyl acetone	–^b^	–^b^	0.15 ± 0.05 ^a^	0.15 ± 0.04 ^a^
alpha-Ionone	–^b^	–^b^	0.14 ± 0.04 ^a^	– ^b^
**Aromatics**				
Naphthalene	0.19 ± 0.05 ^b^	0.07 ± 0.03 ^c^	0.23 ± 0.02 ^b^	0.50 ± 0.11 ^a^
4-Ethyl-2-methoxyphenol	27.75 ± 2.56 ^a^	2.40 ± 0.31 ^b^	– ^c^	– ^c^
4-Ethylphenol	9.83 ± 1.15 ^a^	0.71 ± 0.14 ^b^	0.10 ± 0.04 ^b^	0.11 ± 0.06 ^b^
4-Hydroxy-3-methoxystyrene	1.55 ± 0.40 ^b^	0.63 ± 0.07 ^b^	7.55 ± 1.27 ^a^	13.00 ± 2.86 ^a^
2,4-di-tert-butylphenol	4.78 ± 1.29 ^b^	2.46 ± 0.59 ^c^	4.32 ± 0.78 ^b^	7.29 ± 1.48 ^a^
2,3-Dihydrobenzofuran	1.99 ± 0.09 ^c^	0.78 ± 0.04 ^c^	4.09 ± 0.48 ^b^	7.13 ± 1.81 ^a^

^1^ Values listed includes the mean ± standard deviation from triplicate analysis and triplicate fermentation; ^a,b,c,d^ Statistical analysis by a one-way ANOVA (*p* < 0.05) with same letters showing no significant difference; – = not detected.
